# Serous Cystadenoma: A Review on Diagnosis and Management

**DOI:** 10.3390/jcm12237306

**Published:** 2023-11-25

**Authors:** Kylie Ning, Ashley Salamone, Lindsey Manos, Kelly J. Lafaro, Elham Afghani

**Affiliations:** 1 Division of Gastroenterology, Department of Medicine, Johns Hopkins University School of Medicine, Baltimore, MD 21205, USA; kning2@jh.edu (K.N.);; 2 Division of Surgery, Johns Hopkins University School of Medicine, Baltimore, MD 21205, USAklafaro1@jhmi.edu (K.J.L.)

**Keywords:** serous cystadenoma, pancreatic cysts, CT, MRI, EUS, surveillance, surgical resection

## Abstract

Incidental pancreatic cysts are highly prevalent, with management dependent on the risk of malignant progression. Serous cystadenomas (SCAs) are the most common benign pancreatic cysts seen on imaging. They have typical morphological patterns but may also show atypical features that mimic precancerous and cancerous cysts. If a confident diagnosis of SCA is made, no further follow-up is warranted. Therefore, a preoperative distinction between SCA and precancerous or cancerous lesions is critically essential. Distinguishing an SCA from other types of pancreatic cysts on imaging remains a challenge, thus leading to misdiagnosis and ramifications. This review summarizes the current evidence on diagnosing and managing SCA.

## 1. Introduction

Pancreatic cysts are becoming more prevalent with the increased utilization and advancement in high-quality cross-sectional imaging. A recent real-world data study reported a standardized prevalence of 1.84% [1]. However, population-based studies have reported a prevalence of up to 49.1% with prevalence increasing with age [2,3]. There are three types of pancreatic cysts: benign, precancerous, and cancerous.

Serous cystadenomas (SCAs) are the most common benign lesions, representing 16% of all pancreatic cysts [4]. They were first described in 1978 by Drs. Compagno and Oertel investigating the clinicopathology of 41 pancreatic cyst lesions [5]. They are non-mucinous as opposed to precancerous mucinous cysts known as intraductal papillary mucinous neoplasms (IPMNs) and mucinous cystic neoplasms (MCNs), which are more prevalent. SCAs are clear-cell adenomas rich in glycogen cytoplasm thought to arise from centroacinar cells. They are microscopically described as having a rich capillary network that can help distinguish them from mucinous cysts [6]. Sporadic SCAs are common in patients with VHL syndrome, which affect 1 in 36,000 births. However, those without VHL are also commonly found to have SCAs.

**Figure 1:**
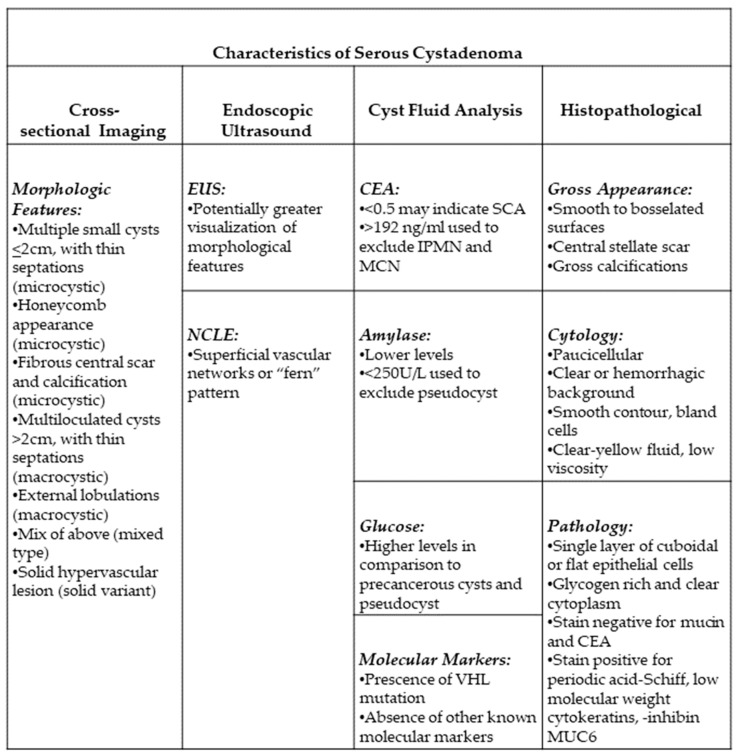
Summary of diagnostic features of SCA. CEA: carcinoembryonic antigen; EUS: endoscopic ultrasound; IPMN: intraductal papillary mucinous neoplasm; MCN: mucinous cystic neoplasm; MUC6: Mucin 6; NCLE: confocal laser endomicroscopy; SCA: serous cystadenoma; VHL: Von Hippel Lindau.

Given the high prevalence of pancreatic cysts, it is important to differentiate among the different types. The challenge lies in diagnosing benign from precancerous or cancerous cysts, as the management is drastically different. Benign cysts are not surveilled given their nature, whereas cancerous cysts are typically recommended to undergo surgery. On the other hand, precancerous cysts are either monitored or referred for surgical resection depending on their risk of advanced neoplasia. Therefore, an accurate diagnosis is the most important first step when evaluating a patient with pancreatic cyst(s). While surgical histopathology is the gold standard to diagnosing any lesion, this is not recommended since pancreatic surgery is associated with significant morbidity and mortality, including long-term complications from the metabolic and digestive effects of pancreatic resection. Clinicians are faced with the challenge of trying to make an accurate diagnosis based on clinical and radiographic features. This review outlines the diagnosis and management of SCAs. Figure 1 summarizes the diagnostic features of SCAs.

## 2. Presentation

Approximately 60–75% of SCAs are found in women in the fifth to seventh decades of life [4,7,8], although there is a wide age distribution. A majority of SCAs arise from the body and tail of the pancreas, while 40% arise from the pancreatic head. Furthermore, approximately 60% of patients with SCAs are asymptomatic and will likely continue to be asymptomatic. A multicenter study with 2622 patients found 61% were asymptomatic at diagnosis. Of the ones who were symptomatic, 27% had non-specific abdominal pain; 9% had pancreaticobiliary symptoms; 5% had diabetes mellitus; and 4% had other symptoms such as abdominal mass, fatigue, nausea, and vomiting [4]. Larger SCAs > 4 cm are more likely to be symptomatic [9] and aggressive [10]. Aggressive SCAs are defined as those with direct invasion into large blood vessels, nerves, lymph nodes, and nearby structures [10]. Symptoms, except for jaundice, are not correlated with the location of SCA [8]. Symptoms and tumor growth rate are indicators of surgical resection. In patients who underwent serial imaging for SCA, lesions greater than 4 cm had a rate had a growth rate of 1.98 cm/year and doubling time of 0.64 years as opposed to tumors less than 4 cm, which had a growth rate of 0.12–0.48 cm/year and doubling time of 2.84 years [9]. In the most extensive cohort study, including 2622 radiographically or histologically suspected SCAs, 0.1% developed serous cystadenocarcinomas [4].

## 3. Diagnosis

### 3.1. Morphologic Features of SCA

Imaging plays a vital role in differentiating pancreatic cysts. Certain features allow for SCA to stand out from other pancreatic cysts. For example, SCAs do not communicate with the pancreatic duct as opposed to IPMNs. SCAs also have four distinct morphological patterns: microcystic, macrocystic, a combination of microcystic and macrocystic, and solid. Microcystic SCAs demonstrate multiple small cysts less than 2 cm in size separated by thin septations. This particular morphology is seen in approximately 50% of SCAs. These lesions may also have a fibrous central scar and calcification seen in up to 30% of microcystic SCAs, giving them a “sunburst” appearance. CT may show a circumvascular sign, which is a result of its hypervascularity. A recent study evaluated the CT features of 71 patients with pancreatic cysts, of which 30 were SCAs, 21 with MCNs, and 20 with branch duct IPMNs who subsequently underwent resection with confirmed surgical pathology, finding that the presence of a central scar or calcification and/or circumvascular sign is 97–100% specific for SCA but has a sensitivity ranging from 23.3 to 76.7%. The presence of the circumvascular sign alone has the highest sensitivity of the three [11]. Microcystic SCAs may also have a honeycomb appearance, indicating multiple tiny cysts.

Macrocystic SCAs, also known as oligocystic patterns, consist of multiloculated cysts >2 cm separated by thin septations. This pattern accounts for approximately 30% of all SCAs. They lack a central scar but demonstrate external lobulations similar to microcystic SCA [4,12,13]. This pattern is more likely found in the head and may cause symptoms such as jaundice or obstruction of the common bile duct [14]. Furthermore, macrocystic pattern SCAs are most easily confused with other pancreatic cysts and are challenging to differentiate. A multicenter study showed that 31% of macrocystic SCAs were misidentified as IPMN, PanNet, MCN, pseudocysts, or ductal adenocarcinomas [15]. Approximately 20% of SCAs consist of microcystic and macrocystic morphology.

The solid variant of SCA is depicted by a solid hypervascular lesion with or without cystic lesions. This pattern accounts for 5% of all SCAs. On histopathology, the cells are arranged in nests, sheets, and trabeculae, separated by thick fibrous bands. The solid lesion within an SCA may also indicate intratumoral hemorrhage, similar to a pancreatic neuroendocrine tumor; solid pseudopapillary neoplasm; or metastatic renal cell carcinoma [4,12,13,16]. The former is significantly smaller when comparing solid SCA to a neuroendocrine tumor and shows wash-in and wash-out enhancement patterns [17].

Despite having distinct features that may distinguish them from other cysts, there may be atypical features such as the presence of parenchyma atrophy, distal location to the lesion, dilation of the upstream pancreatic duct from mass effect, vascular invasion, and invasion of adjacent structures [13,18]. This wide range of morphologies in imaging poses a diagnostic dilemma as it mimics precancerous and cancerous lesions.

### 3.2. Cross-Sectional Imaging

**Figure 2:**
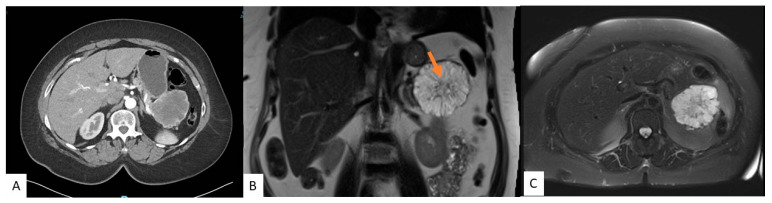
A 54-year-old female with an incidental pancreatic cyst. CT image showing a heterogeneously enhancing lesion with numerous small internal cysts in the tail of the pancreas measuring 6.4 cm × 7.3 cm × 5.4 cm (AP × TV × CC) (**A**). MRI coronal (**B**) and axial (**C**) views of a microcystic lesion in the tail of the pancreas with enhancing septations and central scar (arrow).

Traditionally, CT scans have been used in diagnosing cysts, but increasingly, MRIs are becoming more popular. CT and MRI scans can identify the typical characteristics distinguishing SCAs from other cysts. However, each modality has its advantages and disadvantages. MRI is superior to CT when identifying septations, the presence of a solid component, pancreatic ductal communication, and magnification of the macrocystic nature on T2-weighed images [19,20]. In contrast, CTs can depict calcifications and hypervascularity but provide insufficient soft tissue contrast and spatial resolution, which does not allow for the identification of the microcystic appearance and SCAs less than 2 cm [13,16,18]. Figure 2 shows features of SCA on CT and MRI. Moreover, CT is quick and available in many places, whereas MRIs may not be available, are prone to motion artifacts, take much longer in the scanner, and are more expensive. Recently, there has also been concern about gadolinium deposits in the brain [21]. In blinded studies, radiologists could accurately diagnose SCAs between 23 and 82% [22,23,24]. Given the limitations, combining the two imaging modalities may provide better characterization. Regardless, SCAs often cannot solely be diagnosed based on cross-sectional imaging, and further evaluation is needed.

Radiomics-based approaches and deep learning have been developing in the era of artificial intelligence. They use various images and extract clinical data using an algorithm to predict the type of cyst. In a 2019 study, artificial intelligence provided sensitivity and specificity of 91.9% and 92.9% respectively, in differentiating benign from precancerous cysts [25], although this was limited by a small sample size. The use of specific radiomic features have shown to be superior to standard radiologic features in diagnosing SCA [26]. A recent study showed radiomic-based approaches have an equivalent performance as an academic radiologist with more than 25 years of experience [27].

18F-FDG PET/CT has shown to be promising in the preoperative diagnosis of pancreatic cysts while it is currently not recommended in guidelines [28,29,30]. It relies on the uptake of glucose metabolism by cancer cells. Sperti and colleagues were the first to assess its reliability in differentiating benign from malignant cysts [28]. In later studies, they assessed the reliability of 18F-FDG PET/CT in determining benign cysts from IPMNs. A recent comparison showed that 18F-FDG PET/CT has a diagnostic accuracy of 94% in diagnosing malignant from benign cysts compared to 77% for multidetector CT and 87% for MRI. This study was a prospective single-center study of 31 pancreatic cysts, of which 22 underwent surgical resection with proven histopathology, 4 of which were confirmed SCAs [31] This was in contrast to an earlier retrospective study of 68 patients who underwent 18F-FDG PET/CT, of which 21 underwent surgical resection. The latter study found the sensitivity and specificity to be 57% and 85%, respectively [32]. However, 18F-FDG PET/CT is prone to false positive results, especially with pancreatitis. Further large cohort studies are needed to show the efficacy of 18F-FDG PET/CT in differentiating SCAs from precancerous or cancerous cysts.

### 3.3. Endoscopic Ultrasound

**Figure 3:**
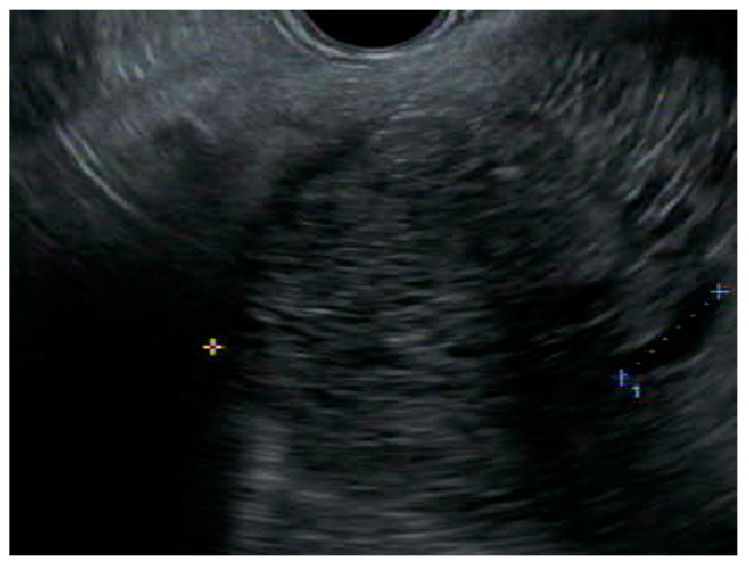
Endoscopic ultrasound image of a microcystic anechoic structure in the body of the pancreas measuring 7 cm × 7.1 cm that was well defined, multi chambered, with thin walls and septations.

Endoscopic ultrasound (EUS) has become more widely utilized as a diagnostic modality in differentiating pancreatic cysts as benign, precancerous, or cancerous, with a diagnostic accuracy of 50–75% [33]. Its high-resolution imaging can delineate classic SCA features, such as the honeycomb feature of multiple small microcysts and multiple compartments [34] (Figure 3). However, it is affected by variable interobserver agreement and is operator dependent. A large study looking at interobserver agreement between endosonographers found that out of all the cysts, only SCAs were met with some certainty in diagnosis. The agreement between endosonographers for SCAs was considered moderately good while the agreement was only considered to be fair for the other types of cysts. Despite this, only 47% of the SCAs were correctly identified [35]. Another major limitation is that EUS is not particularly useful in distinguishing between precancerous cysts, such as IPMN or MCN, and macrocystic SCAs [36]. This is an issue, as misdiagnosis could lead to a drastically different treatment plan. EUS and fine needle aspiration (FNA) is a more valuable tool when compared to EUS alone in diagnosing a pancreatic cyst, having a higher sensitivity and specificity (97% and 100%, respectively) in determining if a cyst should be resected [37].

### 3.4. Cyst Fluid Analysis

Cyst fluid drawn during EUS-FNA is useful in narrowing down a diagnosis for pancreatic cysts based on multiple components, such as cytological features or the presence of mucin, CEA, glucose, amylase, and molecular markers. Cyst fluid analysis has proven useful over the past years. It is increasingly used in various ways as technology improves and costs and turnaround times for analysis decrease, yet there is one major limitation. Most components of cyst fluid analysis have varied cutoffs and no set guidelines for what values may correlate to what specific type of cyst; however, studies have shown that value ranges (higher vs. lower) may be useful in distinguishing broadly between benign and precancerous cysts. Therefore, many components of cyst fluid analysis discussed may not be able to differentiate for SCA specifically but can still be useful in determining management. Another larger limitation is that microcystic SCAs are not amendable to cyst fluid analysis given the multiple tiny cysts.

On gross appearance, cyst fluid is usually clear-yellow and has low viscosity due to the absence of mucin when compared to mucinous cysts such as IPMNs and MNCs [38]. The presence of neoplastic cuboidal epithelium with abundant cytoplasm on cytology is specific for SCA but is only seen in 10% of cases [39]. Typically, they are unicellular to acellular with a clear or hemorrhagic background due to the highly vascular epithelium and highly vascularized fibrous septa. Cell nuclei are usually observed to be centrally located with smooth nuclear contours, a round to oval shape, have indistinct nucleoli, and have evenly distributed chromatin. Other typical characteristics include a lack of atypia, mitotic activity, and necrosis [39,40]. Some literature suggests that the presence of hemosiderin-laden macrophages may be a good surrogate marker for SCA as well. However, it is rare for cytology results to show histiocytes with hemosiderin [36,39]. Unfortunately, there are many challenges posed with obtaining a clear diagnosis from cytology, one being that many procedures such as transduodenal and transgastric approaches during EUS FNA sometimes cause GI contamination and background mucin, leading to misdiagnosis of the SCA as a mucinous cyst such as IPMN or MCN. Additionally, SCAs are occasionally mistaken for mucinous cysts with atypia due to degenerative gastric and intestinal epithelium exhibiting some cellular atypia [39]. These challenges in addition to SCA’s low cellularity and lack of defining characteristics make relying on cytology alone difficult. Thus, results should be considered in combination with CEA, immunostaining, or other measures.

Cyst fluid carcinoembryonic antigen (CEA) analysis is widely available. It can discriminate between benign and precancerous cysts. Still, due to the lack of correlation between CEA levels and dysplasia or cyst growth, CEA values cannot indicate much besides whether a cyst is mucin producing [41]. CEA < 0.5 may be indicative of a benign cyst such as SCA or pseudocyst, whereas >192 ng/mL may indicate a precancerous cyst such as IPMN or MCN with a specificity of 84% and sensitivity of 73% [42]. However, pancreatic neuroendocrine tumors may also have a low CEA.

More recently, cyst fluid analysis of glucose is becoming favorable as it is rapid, widely available, and inexpensive. Since glucose is a common metabolite found in pancreatic cyst fluid, glucose tests can also provide information about cyst types. Precancerous cysts have lower glucose levels due to the hypothesis that glycolysis is the primary cellular ATP source in cancer cells in conjunction with the known finding that pancreatic tumor cells require high glucose levels for metabolism [43]. However, studies have used variable cutoff values with varying results. A recent systematic review and meta-analysis showed intracystic glucose ≤50 mg/dL has a pooled sensitivity and specificity of 90.5% and 88%, respectively [44]. Furthermore, there is variability in glucose testing, whether by colorimetry in a lab or bedside glucometer. Further studies are needed to validate the correlation between techniques.

In a meta-analysis comparing glucose to CEA, glucose testing had significantly higher sensitivity and diagnostic accuracy than CEA alone (91% vs. 56% and 94% vs. 85%, respectively) in differentiating between precancerous cysts, such as IPMNs and MCN, and non-mucin-producing cysts, such as SCA and pseudocyst [45]. Other studies have also shown similar findings [43]. Park and colleagues found SCAs had higher median glucose concentrations when compared to pseudocysts (98 mg/dL and 23 mg/dL, respectively) [46]. While this proves promising at the moment, very few studies have found glucose levels for the detection of specific types of cysts, as Park and colleagues have shown. Given the widespread availability, cheaper cost, and quick turnaround time of glucose testing, it has the potential to be useful in excluding the diagnosis of IPMNs or MCNs. However, while glucose may be useful for differentiating between benign and precancerous cysts, values need to be further examined to see if glucose testing can diagnose a cyst as an SCA vs. other benign cysts specifically.

Cyst fluid amylase, a digestive enzyme found in the pancreas, is a valuable marker suggestive of ductal communication [47]. As expected, amylase is typically very low because SCAs do not communicate with the pancreatic duct [47]. While amylase can be useful in distinguishing between benign vs. precancerous cysts and even between MCNs and IPMNs, currently, there are no specific values that may determine SCAs specifically [47]. Amylase levels of <250 U/L may be indicative of an SCA, IPMN, MCN, or pancreatic neuroendocrine tumor with a sensitivity of 44% and specificity of 98% and are thus only helpful in excluding pseudocysts [39]. Many studies exclude pseudocysts when looking at amylase since it is expected that the amylase levels are high due to the nature of pseudocysts accumulating pancreatic digestive juices [48], but it is assumed that amylase levels >250 U/L are likely to be pseudocysts. Interestingly, one study found that by analyzing cyst fluid from their cohort with a CEA <30, they discovered that amylase <350 could detect 85% of SCA [39].

The use of molecular markers is becoming increasingly popular. While the other components of cyst fluid analysis mentioned do not have concrete values and ways to distinguish between specific types of cysts, molecular markers offer the advantage of indicating specific types of cysts, commonly ones such as IPMN, MCN, or SCA. The presence of a VHL mutation and the absence of a KRAS, GNAS, or RNF43 mutation has a 71–100% sensitivity and 91–100% specificity of correctly identifying SCA preoperatively [49,50]. In a recent study, a VHL mutation had 100% PPV and 98% NPV of SCA. Furthermore, the presence of VHL with TP53 or TERT promoter mutations correlated with SCAs with interval growth in size [50]. While there is great promise in studies showing that SCAs do not have mutations in any other genes but VHL when conducting a molecular markers panel and that VHL can be highly specific and sensitive for diagnosing SCA, there is a caveat [50,51]. Pancreatic neuroendocrine tumors (PanNets) arise in 8–17% of patients with VHL disease and also may account for about 25% of PanNets that are analyzed for molecular markers [51,52]. Patients with SCAs and VHL mutations may actually have a mixed serous-neuroendocrine tumor [51]. Therefore, to avoid confusion of a diagnosis between PanNet and SCA in those who are found to have a VHL mutation, other diagnostic tools should be used in combination.

Unfortunately, not all SCAs arise from VHL mutations and therefore they will not have any known molecular markers. However, the diagnosis of SCA can be made based on the absence of other markers, for example, the absence of the CTNNB1 mutation, which is specific for solid pseudopapillary, lack of KRAS or GNAS mutations, being specific for a mucinous cyst, as well as the use of other advanced endoscopic techniques to characterize the vascular network of the cyst walls.

### 3.5. *Confocal Laser Endomicroscopy*

**Figure 4:**
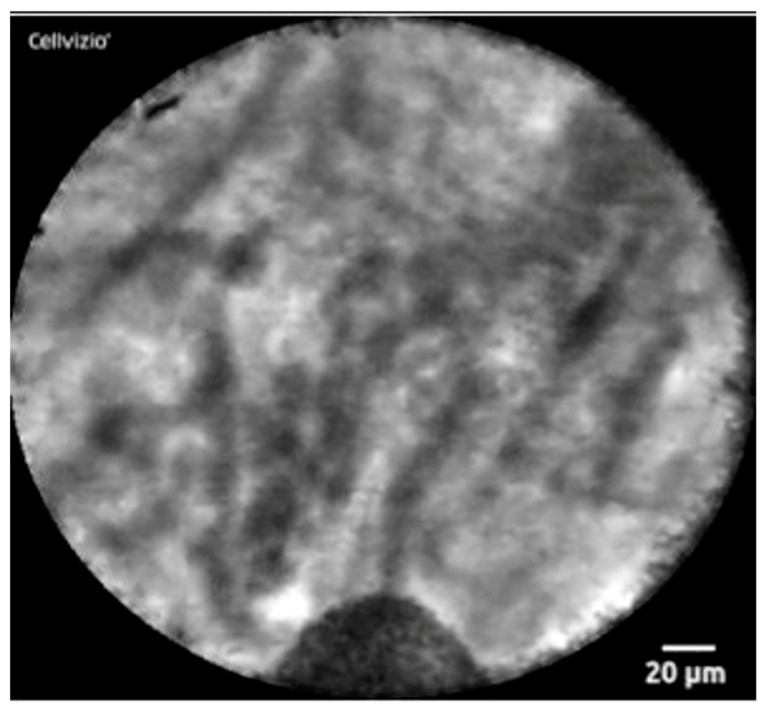
nCLE image of superficial vascular network or “fern pattern”.

Confocal laser endomicroscopy (nCLE) is a technique using real-time visualization of a pancreatic cyst. A miniprobe is inserted through a 19-gauge FNA needle and captures microscopic images of the cyst epithelial lining. It can detect superficial vascular networks or fern patterns, which is specific for SCA. Napoleon and colleagues were the first to describe nCLE in diagnosing SCAs. They highlighted the tightly connected tortuous blood vessels. They may also appear as white vessels on a dark or grey background, or as dark vessels on a clear background, depending on the density of blood cells within the vessels and fluorescein contrast [53] (Figure 4). nCLE has a sensitivity, specificity, and diagnostic accuracy of 99% based on a recent systematic review and meta-analysis, which pooled seven studies. However, nCLE is only available in some places and is operator dependent. It has been reported to have a pancreatitis rate of 1% [54]. Nevertheless, it is possible to distinguish SCA from precancerous cysts with the use of nCLE when all other modalities fail.

## 4. Histopathological Features

In cases where surgery is deemed the best management for suspected IPMN, histopathologic features can confirm that the cyst is an SCA. As mentioned earlier, histopathological features include cells uniquely arranged and separated by thick fibrous bands that may or may not indicate intratumoral hemorrhage [4,12,13,16].

Commonly, the cysts are recognized to be lined by a single layer of cuboidal or flat epithelial cells. In 20–50% of cases, SCAs stain positive for periodic acid-Shiff due to epithelial cells that are commonly glycogen rich and have clear cytoplasm [36,38]. Furthermore, since it is thought that SCAs arise from centroacinar cells, they stain negatively for mucin and CEA among other markers and positive for low-molecular-weight cytokeratins, inhibin, and MUC6 [38,55]

In a study examining 33 surgical cases of histologically confirmed SCAs, it was found that 32 of 33 specimens examined matched the above characteristics, with the cyst being lined by a single layer of cells, with small bland nuclei and clear cytoplasm. In addition, it compared other classical characteristics of being microcystic in pattern, such as being innumerable and irregularly shaped. It also matched the gross appearance characteristics based on this study, including variable-sized cysts separated by thin fibrous septa, smooth to bosselated surfaces, lack of visible papilla, and a visible central stellate scar and calcifications [55].

## 5. Management

In the past, many more SCAs were resected prior to knowledge of the natural history of these lesions. As we have become more aware of the benign course, no further follow-up imaging is indicated. In some cases where the patient is symptomatic, resection may be considered after an informed discussion weighing the risks and benefits of pancreatic resection with the patient. Resection is also performed when the clinician is uncertain whether the cyst is an SCA versus precancerous or cancerous with high-risk or worrisome features.

In a 30 year analysis of pancreatic resections for cysts, 13% of resections were histologically determined to be SCAs. The average tumor size was 42 mm. Most of the patients who underwent resection had abdominal pain (32%), followed by weight loss (10%), pancreatitis (7%), and jaundice (4%). A total of 63% were asymptomatic. However, the trend towards resection of SCAs has significantly decreased from 23% from 1990 to 2000 to 10% from 2011 to 2020 [56]. A recent retrospective analysis of 1488 patients with pancreatic cysts in a prospectively maintained database from 2005 to 2016 showed that 2.1% of surgeries were performed on SCAs. The most common reasons where atypical features on imaging, such as a solid mass, dilation of the main pancreatic duct, enhancing cyst walls, mural nodules, and/or upstream pancreatic atrophy, which are concerning features when suspecting a precancerous cyst and its risk of malignant progression [57].

The management of SCAs is dependent on the confidence of the preoperative diagnosis. Various studies have suggested different approaches to managing SCAs. Tseng and colleagues suggest that those with radiographically consistent SCA should undergo observation with serial CT scans if asymptomatic and/or <4 cm cyst. Although they admit the interval for serial imaging could be more evident, they believe two years to be reasonable. Those with SCAs > 4 cm and/or symptoms should be considered for surgical resection. Their recommendation on surgical resection in asymptomatic SCAs > 4 cm lies with their observation that larger SCAS has more rapid growth and a threefold increase in the likelihood of developing symptoms [9]. However, other studies have found that size and growth rate do not necessarily correlate with symptoms [4,58,59]. Mallelo and colleagues also suggest that a two-year follow-up is warranted, although interestingly, in this study, the observation of 145 patients found a slow growth in SCA over seven years after the baseline [59].

Given the cost and inconvenience of regular imagining, it is worth considering if surveillance imaging should be continued if clinicians are confident in diagnosing SCA based on cross-sectional imaging and other diagnostic tools such as EUS-FNA in asymptomatic patients. Additional research needs to be completed with longer follow-up times before we can truly make this determination regarding the best surveillance strategy. Furthermore, if it is thought that resection is warranted, clinicians should be aware of the risks associated with pancreatic surgery and have an informed discussion with their patients. While pancreatic resection has become much safer over time, it is still associated with morbidity of up to 40% in some series [8,60]. Common complications include pancreatic leaks, exocrine pancreatic insufficiency, and endocrine insufficiency. Patients should be carefully evaluated for fitness prior to resection of these benign cysts.

Guidelines have varying consensus on the management of these lesions. The American College of Gastroenterology (ACG) recommends against follow-up in those with “asymptomatic” SCAs. However, symptoms are not clearly defined [61]. The European guidelines suggest follow-up at 1 year, and surgical resection is only indicated in the presence of symptoms of obstruction (i.e., bile duct, stomach, duodenum, portal vein) [62]. The American College of Radiology (ACR) also recommends no further surveillance for SCAs unless symptomatic, but, like ACG, does not delineate symptoms. They also suggest that asymptomatic SCAs > 4 cm be considered for surgical resection [63].

**Figure 5:**
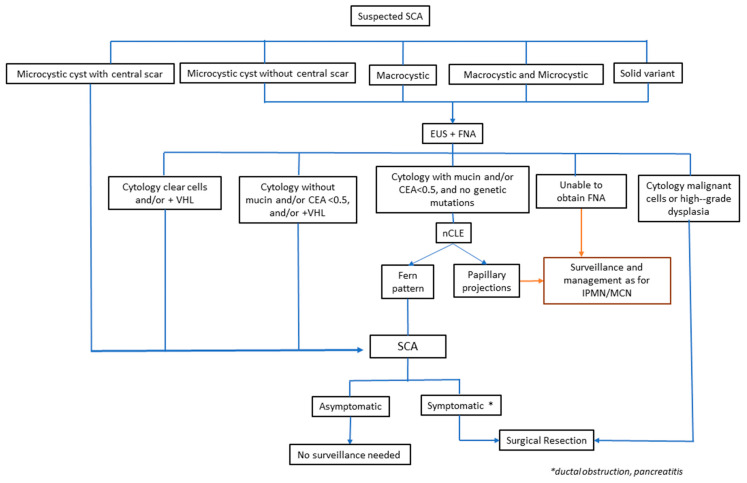
Proposed approach for diagnosis and management of suspected SCAs.

Our approach to suspected SCAs is shown in Figure 5. Pancreatic cysts that are microcystic with a central scar are considered to be SCAs and undergo no further surveillance. However, those suspected SCAs that have other less specific morphologies such as microcystic without central scar, marcrocystic, or solid variant undergo EUS with FNA if feasible. The presence of a VHL mutation or clear cells on cytology is more diagnostic of SCA. In those with lack of these cyst fluid features but with high suspicion for SCA, we perform nCLE to evaluate for fern pattern. If it is not seen, or we are unable to perform FNA, then mucinous cyst is suspected, and continued surveillance for a suspected precancerous lesion is recommended. Those referred for surgical resection include symptomatic SCAs, cytology showing high-grade dysplasia or malignancy for surgical resection, or lack of specific characteristic rendering it to be suspicion for mucinous precancerous cysts with worrisome features.

## 6. Conclusions

SCAs present with a diagnostic dilemma given their multiple presentations. Advancements in cross-sectional and endoscopic imaging as well as molecular markers have allowed us to differentiate SCAs from other cysts better. Their benign nature allows no further follow-up for asymptomatic patients, although no consensus exists currently. Some argue for anywhere from 1–2-year intervals for surveillance, while others argue for no surveillance if asymptomatic. The challenge also lies in definitively diagnosing smaller SCAs where some of the current diagnostic modalities may not be beneficial. As artificial intelligence is being increasingly studied and utilized, the hope is that the diagnosis of SCAs of all sizes can be more definitely made using less invasive approaches. More research needs to be done with long-term follow-ups to create more concise serial imaging protocols. In our practice, if a definitive diagnosis of SCA is made, no further follow-up is needed unless the patient develops symptoms. While SCAs greater than 4 cm have the potential to develop symptoms, size alone is not an indication for resection. Assuming no aggressive or invasive features, no surveillance is warranted if resection is performed.

## Data Availability

Not a applicable.
